# The Kinetochore Proteins Pcs1 and Mde4 and Heterochromatin Are Required to Prevent Merotelic Orientation

**DOI:** 10.1016/j.cub.2007.06.044

**Published:** 2007-07-17

**Authors:** Juraj Gregan, Christian G. Riedel, Alison L. Pidoux, Yuki Katou, Cornelia Rumpf, Alexander Schleiffer, Stephen E. Kearsey, Katsuhiko Shirahige, Robin C. Allshire, Kim Nasmyth

**Affiliations:** 1 Max F. Perutz Laboratories, Department of Chromosome Biology, University of Vienna, Dr. Bohr-Gasse 1, 1030 Vienna, Austria; 2 Research Institute of Molecular Pathology, Dr. Bohr-Gasse 7, 1030 Vienna, Austria; 3 Wellcome Trust Centre for Cell Biology, Institute of Cell Biology, King's Buildings, University of Edinburgh, Edinburgh EH9 3JR, United Kingdom; 4 Department of Zoology, University of Oxford, South Parks Road, Oxford OX1 3PS, United Kingdom; 5 Laboratory of Genome Structure and Function, Division of Gene Research, Center for Biological Resources and Informatics, Tokyo Institute of Technology, 4259 Nagatsuta, Midori-ku, Yokohama 226-8501, Japan

**Keywords:** DNA, CELLCYCLE

## Abstract

**Background:**

Accurate chromosome segregation depends on the establishment of correct—amphitelic—kinetochore orientation. Merotelic kinetochore orientation is an error that occurs when a single kinetochore attaches to microtubules emanating from opposite spindle poles, a condition that hinders segregation of the kinetochore to a spindle pole in anaphase. To avoid chromosome missegregation resulting from merotelic kinetochore orientation, cells have developed mechanisms to prevent or correct merotelic attachment. A protein called Pcs1 has been implicated in preventing merotelic attachment in mitosis and meiosis II in the fission yeast *S. pombe*.

**Results:**

We report that Pcs1 forms a complex with a protein called Mde4. Both Pcs1 and Mde4 localize to the central core of centromeres. Deletion of *mde4^+^*, like that of *pcs1^+^*, causes the appearance of lagging chromosomes during the anaphases of mitotic and meiosis II cells. We provide evidence that the kinetochores of lagging chromosomes in both *pcs1* and *mde4* mutant cells are merotelically attached. In addition, we find that lagging chromosomes in cells with defective centromeric heterochromatin also display features consistent with merotelic attachment.

**Conclusions:**

We suggest that the Pcs1/Mde4 complex is the fission yeast counterpart of the budding yeast monopolin subcomplex Csm1/Lrs4, which promotes the segregation of sister kinetochores to the same pole during meiosis I. We propose that the Pcs1/Mde4 complex acts in the central kinetochore domain to clamp microtubule binding sites together, the centromeric heterochromatin coating the flanking domains provides rigidity, and both systems contribute to the prevention of merotelic attachment.

## Introduction

Individual fission yeast kinetochores, like those of most eukaryotic cells, are associated with multiple microtubules [1]. They therefore run the risk of attaching to microtubules connected to opposing spindle poles, a situation that is known as merotelic attachment [2]. Merotelic attachments occur at high frequencies in the early stages of mitosis but most are corrected. If, however, they persist until anaphase, they cause chromatids to lag on the mitotic spindle, hindering their poleward segregation. Missegregation of lagging chromosomes during anaphase can also be avoided by reducing the fraction of microtubules pulling in the wrong direction [3–6]. Despite correction mechanisms, merotelic kinetochore orientation is a major cause of aneuploidy in mitotic mammalian tissue-culture cells [7]. Very little is known about the molecular mechanisms that prevent or correct merotely. The Aurora B kinase contributes to correction by promoting microtubule destabilization [8, 9]. In fission yeast, the Pcs1 protein has been implicated in preventing merotelic attachment during mitosis and meiosis II. It has been suggested that Pcs1 might clamp together multiple microtubule binding sites on the same kinetochore [10]. It has also been suggested that centromeric heterochromatin, which flanks fission yeast kinetochores, helps to prevent merotelic attachments [11, 12].

Pcs1 is an ortholog of *S. cerevisiae* Csm1, a subunit of the monopolin complex required for the orientation of sister kinetochores to the same pole (mono-orientation) during meiosis I. Surprisingly, Pcs1 is not required for mono-orientation during meiosis I but is required for chromosome segregation during meiosis II and mitosis. Despite the very different phenotypes of *pcs1* and *csm1* mutants, we suggested that the two proteins might nevertheless have similar physiological functions, namely to clamp together microtubule binding sites. Whereas Csm1 is proposed to clamp together microtubule binding sites from sister kinetochores in *S. cerevisiae* that possess only a single site per chromatid, Pcs1 is proposed to clamp together binding sites within the same kinetochore in *S. pombe* [10].

The *S. cerevisiae* monopolin complex consists of two high-affinity subcomplexes, namely Csm1/Lrs4 and Mam1/Hrr25, which associate with each other to form a quaternary complex [10, 13]. If the molecular function of monopolin were conserved during evolution, we would predict proteins homologous to *S. cerevisiae* monopolins to be present in various species. Although Csm1 and Hrr25 have clear orthologs in *S. pombe* [10, 13], we have hitherto failed to identify orthologs of Lrs4 and Mam1 by bioinformatics tools. We therefore adopted a biochemical approach: that is, we purified Pcs1 and used mass spectrometry to identify proteins associated with it.

## Results

### Pcs1 Forms a Complex with Mde4

**Figure 1:**
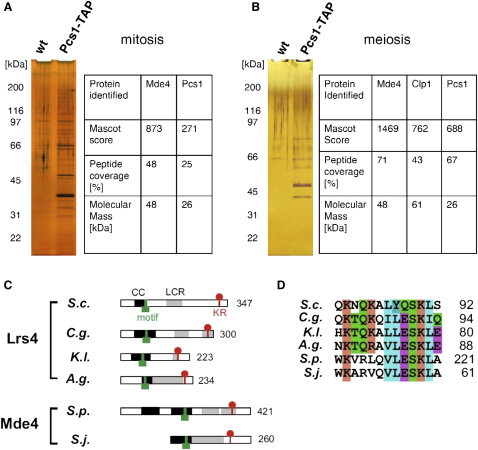
Pcs1 Forms a Complex with Mde4 (A) Proteins associated with TAP-tagged Pcs1 were isolated by tandem affinity purification from cycling mitotic *S. pombe* cells (K12253). Purified proteins were separated on a gel and visualized by silver staining. In parallel, samples were subjected to analysis by tandem mass spectrometry (MS/MS). Proteins that specifically associated with Pcs1-TAP are shown (see Figure S1A for the full list of proteins identified). (B) Proteins associated with TAP-tagged Pcs1 were isolated by tandem affinity purification from diploid *S. pombe* cells induced to enter synchronous meiosis by inactivation of Pat1 (K12524) [15] and harvested around metaphase I. Proteins were analyzed as described in (A). Proteins that specifically associated with Pcs1-TAP are shown (see Figure S1B for the full list of proteins identified). (C) Sequence features of Lrs4 and Mde4 proteins: coiled-coil regions (CC) are indicated by a black box, the low-complexity regions (LCR) are shaded in gray, the motif within the coiled-coil region is indicated by a green box, and the lysine/arginine-rich cluster (KR) at the C terminus is marked by a red circle. *S.c.*, *Saccharomyces cerevisiae*; *C.g.*, *Candida glabrata*; *K.l.*, *Kluyveromyces lactis*; *A.g.*, *Ashbya gossypii*; *S.p.*, *Schizosaccharomyces pombe*; *S.j.*, *Schizosaccharomyces japonicus*. (D) A motif within the coiled-coil region that is present in Lrs4 and Mde4 proteins. *S.c.*, *Saccharomyces cerevisiae*; *C.g.*, *Candida glabrata*; *K.l.*, *Kluyveromyces lactis*; *A.g.*, *Ashbya gossypii*; *S.p.*, *Schizosaccharomyces pombe*; *S.j.*, *Schizosaccharomyces japonicus*.

We used a tandem affinity purification (TAP) strategy [14] to isolate Pcs1 together with associated proteins from cycling mitotic cells as well as from *pat1*-synchronized meiotic cells harvested around metaphase I [15]. Purified proteins were separated on SDS-PAGE gels and silver-stained. In parallel, samples were subjected to analysis by mass spectrometry. Pcs1 associated with high levels of a thus far uncharacterized protein called Mde4 during both mitosis and meiosis as well as with Clp1 (homolog of Cdc14 phosphatase) specifically during meiosis (Figures 1A and 1B). Mde4 (*mei4*-dependent expression 4) is encoded by the SPBC6B1.04 gene, the expression of which depends on Mei4, a forkhead-like transcription factor required for the progression of meiosis [16]. In *S. cerevisiae*, the monopolin Csm1, an ortholog of Pcs1, forms a tight complex with Lrs4 [13], so we asked whether Mde4 might be the fission yeast ortholog of the monopolin Lrs4.

By using iterative PSI-BLAST searches, we detected orthologs of Lrs4 in *Candida glabrata*, *Eremothecium gossypii*, and *Kluyveromyces lactis* (for a multiple alignment of the Lrs4 protein family, see Figure S9 in the Supplemental Data available online). All Lrs4 proteins share an N-terminal coiled-coil region and regions of low complexity C-terminal to the coiled coils (Figure 1C). We could not detect a clear homolog of *S. pombe* Mde4 in the NCBI nr database, but a fragment homologous to Mde4 could be extracted from sequence traces of *Schizosaccharomyces japonicus*. Like Lrs4 orthologs, the two *japonicus* and *pombe* Mde4 proteins are predicted to contain coiled-coil regions at their N termini whereas their C-terminal halves contain low-complexity regions of polar composition. We searched for short conserved motifs within the Lrs4 and Mde4 proteins and identified a motif within the coiled-coil region that is present in all homologs of Lrs4 and Mde4 (Figure 1D). In addition, all homologs of Lrs4 and Mde4 contain a cluster of positively charged residues close to their C termini, which could be a part of a nuclear localization signal [17].

Although we could not detect any significant sequence homology between Lrs4 and Mde4 proteins, we found that they share similar sequence features. Sequencing of other yeast genomes related to *S. pombe* could provide more evidence for a possible phylogenetic relationship between Lrs4 and Mde4. Importantly, our definition of the common features between Lrs4 and Mde4 will help in searches for possible orthologs in higher eukaryotes.

### Pcs1 and Mde4 Localize to the Central Region of Centromeres

**Figure 2:**
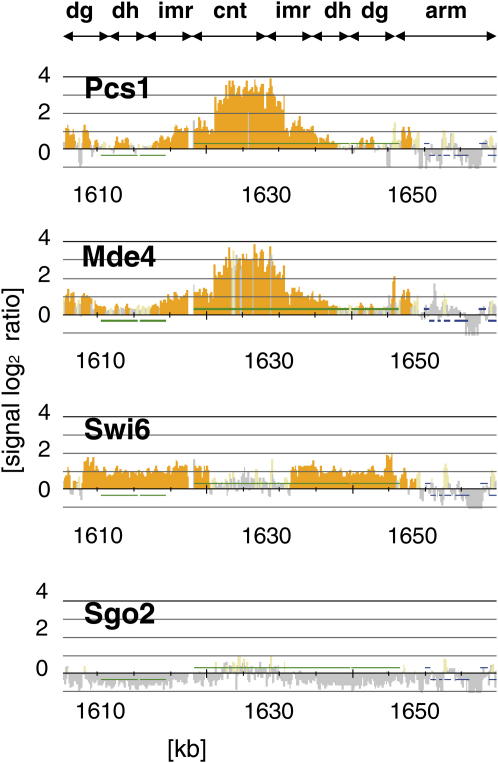
Pcs1 and Mde4 Localize to Centromeres Cycling *S. pombe* cells carrying Pcs1-GFP (K11251), Mde4-GFP (K12417), Swi6-GFP (K13893), or Sgo2-GFP (K12111) were harvested, and the distribution of the GFP-tagged proteins on all three fission yeast chromosomes was analyzed by chromatin immunoprecipitation followed by hybridization to high-density oligonucleotide arrays (ChIP-chip). Only the centromeric region of chromosome 2 is shown (see http://shirahigelab.bio.titech.ac.jp/ccdata/gregan/ for the full set of data). The central region (*cnt*), the innermost repeats (*imr*), and the outer repeats (*dg*, *dh*) of the centromere as well as centromere-proximal region of the chromosome arm (arm) are indicated.

Like Pcs1, Mde4 localized to nuclei during both mitosis and meiosis. Both Pcs1 and Mde4 were enriched in the region of the nucleus that stains poorly with DAPI, which corresponds to the nucleolus. Both proteins were also concentrated in nuclear foci, which presumably represent centromeres (Figure S3). To determine more precisely the chromosomal regions to which Pcs1 and Mde4 bind, we analyzed the localization of Pcs1 and Mde4 along the whole length of the chromosomes by chromatin immunoprecipitation followed by hybridization to high-density oligonucleotide arrays (ChIP-chip) covering all three *S. pombe* chromosomes [18]. We noticed that the ChIP-chip protocol based on PCR amplification of *S. pombe* genomic DNA is prone to biased amplification. We therefore developed a new protocol via a T7-based in vitro transcription linear amplification method that does not require PCR amplification. By using this protocol on material from cycling cells, we confirmed enrichment of the heterochromatin protein Swi6/HP1 at outer centromeric repeats, subtelomeric regions, and the mating-type locus, although we did not detect any significant centromeric signal of Sgo2, which is consistent with previous reports [11, 19, 20] (Figure 2; http://shirahigelab.bio.titech.ac.jp/ccdata/gregan/). The distributions of Pcs1 and Mde4 along all three chromosomes were very similar. Both proteins were enriched at the innermost repeats (*imr*) and central core (*cnt*) regions of centromeres. Although Pcs1 and Mde4 localized to nucleoli (Figure S2), we could not detect binding of Pcs1 and Mde4 to nucleolar chromatin in our ChIP-chip assay (http://shirahigelab.bio.titech.ac.jp/ccdata/gregan/). We speculated that Pcs1 and Mde4 may be tightly bound to centromeres but not to nucleolar chromatin. To test this, we used an in situ chromatin binding assay to determine chromatin association of Pcs1 in single cells [21]. Detergent extraction removed Pcs1 from nucleoli but not from the (one to three) nuclear foci that presumably represent kinetochores (Figure S3). Thus, both ChIP-chip analysis and chromatin binding assay suggest that Pcs1 and Mde4 bind tightly to centromeres but not to nucleolar chromatin. We conclude that Pcs1 and Mde4 bind predominantly to the central core of centromeres. This is consistent with the notion that Pcs1 and Mde4 might clamp together microtubule attachment sites, because other proteins required for proper kinetochore orientation (e.g., Moa1) also associate with the central core of centromeres [22].

Mutations in genes for other centromeric proteins (e.g., Swi6, Clr4, Mis6, Cnp1/CENP-A) alleviate transcriptional silencing at centromeres and allow expression of marker genes placed within the centromeric DNA [23]. In contrast, Pcs1 is not required to maintain the silenced state of marker genes inserted in centromeric chromatin either within the central core or the outer repeats (data not shown). This is consistent with the notion that Pcs1 does not directly affect the structure of centromeric chromatin.

### Pcs1 and Mde4 Are Required for Accurate Chromosome Segregation during Mitosis and Meiosis II

**Figure 3:**
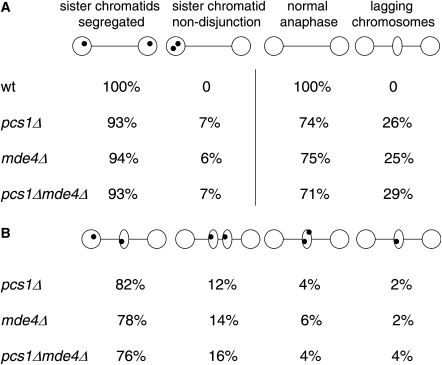
Pcs1 and Mde4 Are Required for Chromosome Segregation during Mitosis (A) Wild-type (K11338), *pcs1Δ* (K14820), *mde4Δ* (K14818), and *pcs1Δ mde4Δ* (K14822) haploid cells expressing *lys1*-GFP were fixed and stained with antibodies against tubulin and GFP [30]. Nuclei were visualized by Hoechst staining. Samples were examined under the fluorescence microscope, and segregation of chromosome I marked by *lys1*-GFP was scored in 100 late anaphase cells. (B) Lagging chromosomes in *pcs1Δ* and in *mde4Δ* cells are single chromatids. Cells were prepared as in (A) and those with lagging *lys1*-GFP signal (representing chromosome I) were scored (n = 50).

Neither *pcs1^+^* or *mde4^+^* are essential genes. However, we observed that strains lacking Pcs1 or Mde4 were hypersensitive to the microtubule-destabilizing drug thiabendazole, which is consistent with the idea that these mutants are defective in the interaction between kinetochores and microtubules (Figure S4). During mitosis, both *pcs1Δ* and *mde4Δ* mutant cells had a high frequency of lagging chromosomes in late anaphase cells, which was associated with a high rate of sister-chromatid nondisjunction (Figure 3A). Sister *lys1* sequences on chromosome I marked by GFP [24] segregated to the same pole in 7% of *pcs1Δ* cells, in 6% of *mde4Δ* cells, in 7% of *pcs1Δ mde4Δ* double mutant cells, but never in wild-type cells. We next determined whether the lagging chromosomes in *pcs1Δ* and *mde4Δ* cells contained one or two chromatids. In the majority of cells with lagging chromosomes containing a *lys1*-GFP signal, its sister *lys1*-GFP signal had already segregated to a pole. This implies that the lagging chromosomes are individual chromatids that have already disjoined from their sisters. In most of the remaining cases, both chromosome I chromatids were lagging and sister *lys1*-GFP signals had clearly disjoined from each other (Figure 3B). We conclude that Pcs1 and Mde4 are essential for accurate chromosome segregation during mitosis and that lagging chromosomes in *pcs1Δ* and *mde4Δ* mutant cells are mostly single chromatids.

Deletion of *pcs1^+^* or *mde4^+^* reduced spore viability, which is presumably caused by missegregation of chromosomes during meiosis (Figure S5). To test this, we scored segregation in a strain in which both copies of chromosome I were marked by GFP close to *cen1* (homozygous *lys1*-GFP). Scoring of the *lys1*-GFP dots in ordered asci suggested that sister chromatids missegregate during the second but not the first meiotic division (Figure S5). To investigate the segregation of sister chromatids directly, we measured segregation in a strain in which only one copy of chromosome I was marked by *lys1*-GFP (heterozygous *lys1*-GFP). As in wild-type cells, sister centromeres in both *pcs1Δ* and *mde4Δ* mutant cells cosegregated to the same pole during meiosis I (Figure S6A). We attribute the rare cases of segregation of sister *lys1*-GFP sequences to opposite poles to recombination taking place between centromere and *lys1*. In wild-type cells, sister *lys1*-GFP sequences invariably segregated to opposite poles of one of the two anaphase II spindles. In *pcs1Δ* and *mde4Δ* mutant cells, sister sequences frequently segregated to the same pole (12% and 8%, respectively). A similar level of missegregation (9%) was observed in *pcs1Δ mde4Δ* double mutant cells, which is consistent with the notion that Pcs1 and Mde4 function in the same biological process (Figure S6B).

These data imply that both Pcs1 and Mde4 are needed for faithful sister-chromatid segregation during mitosis and meiosis II. However, unlike their budding yeast counterparts Csm1 and Lrs4, Pcs1 and Mde4 are dispensable for segregation of homologs during meiosis I. This is consistent with the observation that fission yeast has an additional system ensuring mono-orientation of sister kinetochores during meiosis I [22].

### Lagging Kinetochores in *pcs1, mde4*, and *clr4* Mutants Display Features of Merotelic Attachment

**Figure 4:**
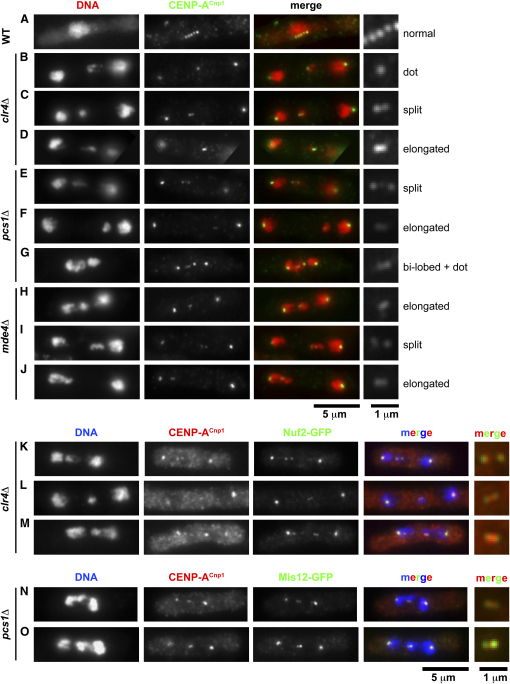
Lagging Chromosomes Have Stretched Kinetochores (A–J) Wild-type (A), *clr4Δ* (B–D), *pcs1Δ* (E–G), and *mde4Δ* (H–J) cells were fixed and processed for immunofluorescence with anti-CENP-A^Cnp1^ antiserum and stained with DAPI to visualize DNA. Merged image is false-colored red for DAPI and green for CENP-A^Cnp1^. (A) Normal round appearance of kinetochores in a wild-type cell in early mitosis. (B–J) The kinetochores on lagging chromosomes displayed a range of morphologies: normal or dot-like (B, G); elongated (D, F, H, J); bilobed shape (G); split (C, E, I). The cell in (G) has two lagging kinetochores, of which one is bilobed and the other is dot-like. Stretched kinetochores are also shown at increased magnification in the panels on the right, along with the classification used in the quantification in Table S1. (K–M) *clr4Δ* cells expressing Nuf2-GFP were fixed and processed for immunofluorescence with anti-GFP and anti-CENP-A^Cnp1^ antiserum, and DNA was stained with DAPI. The merged image is false-colored blue for DNA, green for Nuf2-GFP, and red for CENP-A^Cnp1^. Higher-magnification images of the lagging kinetochores (only Nuf2-GFP and CENP-A^Cnp1^ signals) are shown on the right. (K) A lagging chromosome 3 (distinguished by the trailing rDNA chromatin; the other chromosome 3 is at the right pole) has a split kinetochore (both CENP-A^Cnp1^ and Nuf2-GFP). (L) Lagging chromosome has a slightly elongated CENP-A^Cnp1^ spot, flanked by Nuf2-GFP spots (this cell is probably diploid). (M) Lagging chromosome has a slightly elongated CENP-A^Cnp1^ spot flanked by Nuf2-GFP spots. (N and O) *pcs1Δ* cells expressing Mis12-GFP treated and presented as above. Lagging chromosomes have a slightly elongated CENP-A^Cnp1^ spot, flanked by Mis12-GFP spots.

A key to understanding the molecular function of the Pcs1/Mde4 complex is to discover why individual chromatids fail to segregate to the poles (lag) on anaphase spindles. We have previously shown that neither premature loss of sister chromatid cohesion nor a failure to resolve sister-chromatid cohesion on time are likely to be the causes [10]. Our suggestion that Pcs1 (and Mde4) might be responsible for clamping together microtubule attachment sites on the same kinetochore raises another possibility, namely that chromatids are attached merotelically in the mutants. This might also be the cause of the high frequency of lagging chromosomes caused by *clr4* or *swi6* mutations that affect centromeric heterochromatin [11, 12]. In mammalian, plant, and insect cells, merotelic attachment induces stretching of the kinetochores of lagging chromosomes during anaphase [2, 25, 26]. Sometimes, merotelically attached kinetochores are extensively stretched, creating two domains that appear as separate spots. Importantly, kinetochore stretching of this nature never occurs in normally oriented (amphitelic) kinetochores and it is therefore characteristic of merotelically attached kinetochores [7]. Despite their small size, merotelically attached chromatids might possess similar properties in *S. pombe*. To test this, cycling cells lacking either Pcs1, Mde4, or Clr4 were fixed and processed for immunofluorescence with antibodies against the centromere-specific histone H3 variant CENP-A^Cnp1^, which forms the chromatin platform upon which the rest of the kinetochore is assembled (Figure 4). Fission yeast kinetochores normally possess a round, dot-like appearance (Figure 4A). In contrast, the kinetochores of lagging chromosomes in the mutants displayed a variety of CENP-A^Cnp1^ staining patterns. Many lagging kinetochores had a dot-like morphology that appeared indistinguishable from normal kinetochores, as shown in the example in Figure 4B. However, other lagging kinetochores possessed a CENP-A^Cnp1^ signal that was stretched in the plane of the spindle, examples of which are shown in Figures 4C–4J. These were classified as having an elongated morphology, a bilobed shape, or a kinetochore signal split in two. Stretched (including elongated, bilobed, and split) CENP-A^Cnp1^ signals were observed for 25%–32% of lagging kinetochores in *pcs1Δ*, *mde4Δ*, and *clr4Δ* mutants (Table S1). Very similar images of stretched kinetochores were also observed when the mutants were stained for tagged versions of various other kinetochore proteins such as CENP-C^Cnp3^, Mis12, Nuf2, and Sim4 (data not shown, and below). The diameter of kinetochores (CENP-A^Cnp1^-staining dots) in early mitosis in wild-type cells was 0.41 ± 0.03 μm (Table S2). Dot-shaped lagging kinetochores had similar dimensions to normal kinetochores, whereas stretched kinetochores were elongated in the plane of the spindle approximately 2-fold on average (∼0.8 μm) but maintained a normal width (Table S2). To compare the CENP-A^Cnp1^ chromatin with that of the overlying kinetochore, *pcs1Δ* and *clr4Δ* cells expressing GFP-tagged kinetochore proteins (Mis12-GFP or Nuf2-GFP) were double-stained with antibodies to GFP and to CENP-A^Cnp1^. In the majority of lagging chromosomes, the CENP-A^Cnp1^ and GFP signals were coincident, as observed for the split kinetochore in Figure 4K. Intriguingly, we sometimes observed an elongated CENP-A^Cnp1^ signal flanked by two Mis12-GFP or Nuf2-GFP spots (Figures 4L–4O; split pattern) (in no case were two CENP-A spots seen flanking an elongated GFP signal). This pattern may represent kinetochores in which the underlying CENP-A^Cnp1^ chromatin is merely stretched or elongated, although the overlying kinetochore structure has been split by spindle forces.

**Figure 5:**
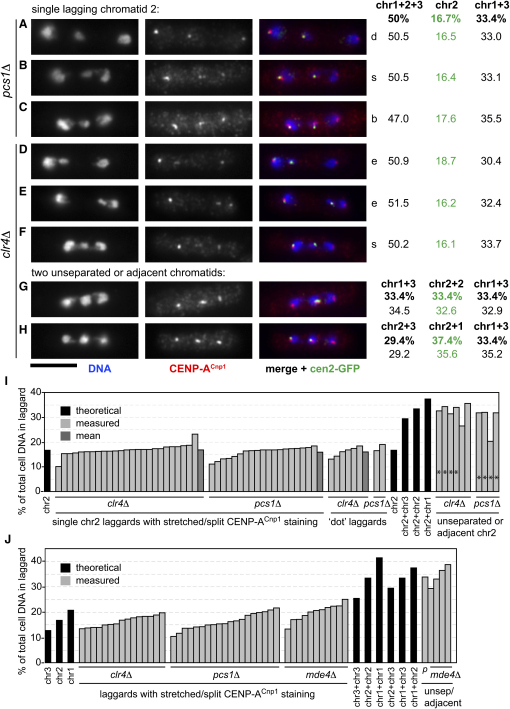
Quantification of DAPI Fluorescence Intensity Demonstrates that Lagging Chromosomes with Stretched Kinetochores Are Single Chromatids (A–H) Immunofluorescence of *pcs1Δ* (A–C) and *clr4Δ* (D–H) cells containing GFP-marked chromosome 2, via anti-GFP antibodies (green; cen2-GFP), anti-CENP-A^Cnp1^ (red), and DAPI (blue; DNA). DAPI and CENP-A^Cnp1^ images (autoscaled) are shown separately and merged together with the anti-GFP image, which was processed to highlight the cen2-GFP spots and diminish the nucleoplasmic background LacI-GFP fluorescence. (A–F) Cells with a single chromosome 2 laggard are shown, displaying either a dot ([A], d) or stretched CENP-A^Cnp1^ signal ([B–F]; s, split; b, bilobed; e, elongated). Quantification of the DAPI fluorescence intensity is presented to the right of the images (numbers are percentages each DAPI-stained object makes to the total DNA in the cell; see text and Supplemental Experimental Procedures for details). Theoretical percentages are shown at the top in bold. The values for chromosome 2 are shown in green. (G) Cell with two unseparated chromosome 2 chromatids lagging. (H) Cell with a single chromosome 2 chromatid adjacent to another chromatid (probably chromosome 1). Theoretical (bold) and measured percentages are shown. Scale bar represents 5 μm. (I) Bar graph of DAPI fluorescence intensity measurements of cen2-GFP-containing cells (as shown in [A]–[H]). Height of bar indicates percentage contribution the lagging chromosome 2 makes to the total DNA in the cell. Theoretical relative sizes of chromosome 2 and combinations of two chromatids are shown in black. Measurements of laggards are shown in light gray, mean for each set shown in dark gray. The proportion of stretched versus dot laggards shown in the graph is not representative of the population; a small number of “dot” laggards are shown for comparison only. Also presented are unseparated/adjacent chromosome 2 laggards (asterisks), and those that presumably represent one chromosome 2 chromatid (the other was at a pole) adjacent to either a chromosome 3 or a chromosome 1 lagging chromatid. (J) Bar graph of DAPI fluorescence intensity measurements of unmarked laggards (as shown in Figure 4). Laggards shown in the main left part of the graph were classified as single chromatids with stretched CENP-A^Cnp1^ staining (Table S1), whereas those at the right had been interpreted as unseparated or adjacent chromatids. p, *pcs1Δ*.

Several observations support the notion that the stretched signals represent single kinetochores rather than two closely opposed sister kinetochores. The vast majority of laggards are single chromatids (Figure 3B, Figure S7), and therefore the kinetochore signals on laggards are not due to unseparated sister chromatids in the majority of cases. In addition, the CENP-A^Cnp1^ (or Mis12-GFP, Nuf2-GFP) signal on stretched kinetochores generally appear less bright than single normal kinetochores, as would be expected if the signal were stretched out over a larger area (Figure 4G). However, despite these arguments, it is formally possible that the stretched/split CENP-A^Cnp1^ signal on any particular lagging mass of DNA is instead due to unseparated sister chromatids or adjacent nonsister chromatids. To exclude these possibilities, we measured fluorescence intensity of DAPI-stained chromatin to provide estimates of the relative masses of chromosomal DNA entities within mitotic cells. The haploid fission yeast genome of 13.8 Mb is divided between chromosomes 1 (5.7 Mb), 2 (4.6 Mb), and 3 (3.5 Mb). A mitotic cell (2n DNA content) contains 27.6 Mb, and the contribution each chromatid makes to this total is 20.7% for chromosome 1, 16.7% for chromosome 2, and 12.7% for chromosome 3. Any combination of two chromatids would account for 25.4%–41.4% of the total mass of DNA in the cell (see Table S3 for details, Figure 5). We reasoned that it should be possible to determine whether a laggard is a single chromatid or two chromatids on the basis of its relative mass in the cell. As proof of principle, and to determine whether fluorescence intensity is proportional to DNA mass, we initially examined images of *clr4Δ* cells containing a lagging GFP-marked chromosome 2 (cen2-GFP; *lac* operator array inserted 5 kb from *cen2*) [27]. Because the majority of these cells have a single lagging chromosome 2 chromatid, with its sister's GFP dot at a pole, the predicted relative masses are: 50% (chromosomes 1+2+3 on one pole), 33.4% (chromosomes 1+3 on the other pole), and 16.7% (chromosome 2 laggard). The values determined by quantification of DAPI fluorescence were very similar to the theoretical values: 49.0% ± 2.1%, 33.0% ± 2.2%, and 17.8% ± 1.6% (mean ± SD, n = 16) (Figure S10 and data not shown). In addition, for the small number of cells containing unseparated or adjacent lagging chromosome 2s, measurement of DAPI fluorescence also produced values close to the theoretical value for two chromatids (33.4%; Figure S10). Thus, these analyses demonstrate that quantification of DAPI fluorescence intensities is a valid method for estimation of relative DNA mass.

We next examined the morphology of kinetochores on laggards that were demonstrably single chromatids, by CENP-A^Cnp1^ staining of *clr4Δ* and *pcs1Δ* strains containing cen2-GFP. Cells with one cen2-GFP dot at a pole and one cen2-GFP dot lagging (i.e., a single chromosome 2 chromatid) were analyzed for evidence of kinetochore stretching/splitting. Approximately one-third of chromosome 2 laggards had stretched/split CENP-A^Cnp1^ signals (15/50 cells for *clr4Δ* and 17/50 cells for *pcs1Δ*) (Figure 5), similar to the proportion observed for unmarked chromosomes (Table S1). Cells with a stretched/split CENP-A^Cnp1^ signal on a chromosome 2 laggard were analyzed by quantification of DAPI staining. In virtually all cases (Figures 5B–5F and 5I), the proportion of DAPI signal in the chromosome 2 laggard was consistent with it being a single chromatid (theoretical: 16.7%; *clr4Δ*, measured: 16.8% ± 2.1% [n = 25]; *pcs1Δ*, measured: 15.9% ± 2.1% [n = 19]; Figure 5I). A small number of chromosome 2 laggards with nonstretched (dot) kinetochore morphology were also analyzed for comparison (Figures 5A and 5I); their relative DAPI fluorescence indicated that, as expected, they too are single chromatids. In addition, we analyzed rare cases of unseparated chromosome 2 s or those with a large mass of lagging DNA that included one chromosome 2 laggard (Figures 5G–5I); 9 out of 10 were found to contain DAPI fluorescence signal consistent with two chromosome 2s or one chromosome 2 plus either chromosome 1 or chromosome 3. Thus, we confirmed by DAPI quantification that chromosome 2 laggards with stretched kinetochores are indeed single chromatids.

In addition, we performed DAPI quantification on images of *clr4Δ*, *pcs1Δ*, and *mde4Δ* cells containing three untagged chromosomes (i.e., cells shown in Figure 4). The chromosomes are unmarked, so the identity of laggards and the segregation pattern of chromatids are not known. It should also be noted that the relative mass of a single chromosome 1 (20.7%) is reasonably similar to that of two chromosome 3 chromatids (25.4%), although all other single versus double combinations have very different theoretical masses (Table S3). Despite these caveats, the relative DNA masses of laggards with stretched or split kinetochore signals indicated that the majority of laggards were indeed single chromatids (Figure 5J).

Thus, the use of DAPI fluorescence intensity quantification to determine the relative mass of laggards confirms our contention that laggards with stretched/split kinetochores are single chromatids rather than any combination of two or more chromatids. This strongly supports our proposal that lagging chromosomes in *clr4Δ*, *pcs1Δ*, and *mde4Δ* mutants are merotelically attached.

**Figure 6:**
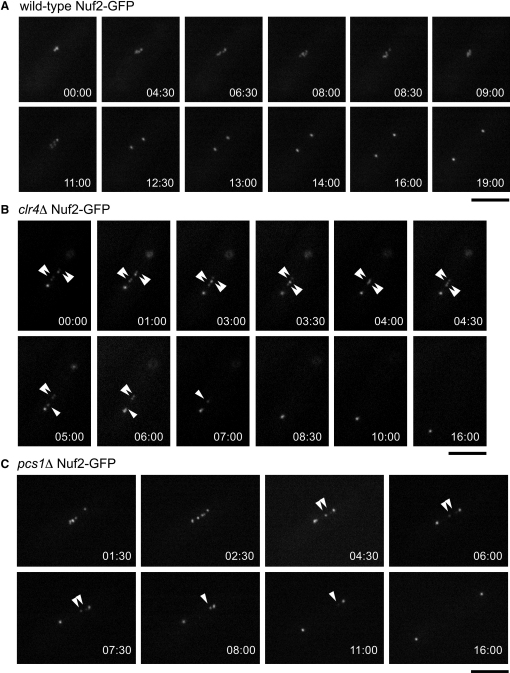
Live Analysis of Lagging Kinetochores Wild-type (A), *clr4Δ* (B), or *pcs1Δ* (C) cells expressing Nuf2-GFP were filmed (Movies S1, S2, and S7). Selected frames are shown; times indicated are in minutes. Arrowheads indicate positions of lagging kinetochores: single arrowhead indicates a dot-shaped or elongated kinetochore; double arrowhead indicates a split kinetochore. Scale bars represent 5 μm.

To examine further the properties of lagging kinetochores in *pcs1Δ* and *clr4Δ* mutant cells, we analyzed kinetochores marked by Nuf2-GFP by live cell imaging (Figure 6, Figure S8, and Supplemental Movies). Live movies confirmed that in both *pcs1Δ* and *clr4Δ* mutant cells lagging kinetochores often displayed stretching or splitting, when they were in between the spindle poles but frequently rounded up when they were reaching one of the poles (Figure 6, Figure S8, and Supplemental Movies). We interpret this as follows: variation in tension across the merotelically attached kinetochore causes changes in the degree of splitting of the kinetochore; the two halves of the kinetochore recoil and are reunited perhaps when microtubule connections with one pole are broken, which allows movement of the lagging chromosome to the pole. The behavior of lagging kinetochores in live cells supports the notion that lagging kinetochores in fission yeast are merotelically attached.

## Discussion

Pcs1 is an *S. pombe* kinetochore protein that is homologous to the monopolin subunit Csm1 in *S. cerevisiae*. Like Csm1, which binds tightly to Lrs4, we show here that Pcs1 forms a complex with a protein called Mde4. Even though Mde4 and Lrs4 do not share any obvious sequence identity, several lines of evidence are consistent with the notion that Mde4 is the fission yeast counterpart of Lrs4. First, both Csm1/Lrs4 and Pcs1/Mde4 complexes copurify with Cdc14 phosphatase [13]. Second, all four proteins (Csm1, Lrs4, Pcs1, Mde4) share similar localization patterns, being concentrated at kinetochores and within nucleoli. Whereas Pcs1 and Mde4 associate with kinetochores (and nucleoli) throughout the mitotic cell cycle, Csm1 and Lrs4 associate with kinetochores only during meiosis I. Lastly, Lrs4 and Mde4 share a similar distribution of sequence features and motifs.

The notion that Csm1/Lrs4 and Pcs1/Mde4 are equivalent complexes must nevertheless explain the very different phenotypes caused by their inactivation. Inactivation of the Csm1/Lrs4 complex causes a defect in chromosome segregation during meiosis I whereas inactivation of Pcs1/Mde4 has little or no effect on meiosis I but causes missegregation of chromosomes during mitosis and meiosis II. Our conclusion that Csm1/Lrs4 facilitates mono-orientation of sister kinetochores during meiosis I [10] whereas Pcs1/Mde4 prevents merotely during mitosis and meiosis II (this study) suggests that the two complexes may in fact have fundamentally the same physiological function, namely to clamp together kinetochore microtubule binding sites. *S. cerevisiae* kinetochores bind only a single microtubule, at least during mitosis, and coordination between multiple microtubule binding sites is required only during the first meiotic division when sister kinetochores must behave as a single unit and attach to microtubules from the same pole. *S. pombe* kinetochores, in contrast, bind multiple microtubules at all stages of their life cycle and therefore require a mechanism to hinder the attachment of kinetochores to microtubules from different poles [1]. We suggest that the *S. cerevisiae* Csm1/Lrs4 complex adopts a function similar to that of Pcs1/Mde4 only during meiosis I when the former is released temporarily from the nucleolus. This hypothesis explains why Csm1/Lrs4 is not required for mitosis in *S. cerevisiae*. Because of their unitary kinetochores, merotely cannot arise in this organism. However, our hypothesis does not explain per se why Pcs1/Mde4 is unnecessary for chromosome segregation during the first meiotic division in *S. pombe*. To explain this, we suggest that the function performed by Pcs1/Mde4 is undertaken during the first meiotic division by proteins such as Moa1 [22] that might suppress merotely as well as promote mono-orientation of sister kinetochores.

The conclusion that the Pcs1/Lrs4 complex prevents merotely is based on the finding that *pcs1Δ* and *mde4Δ* mutants have a high frequency of lagging chromosomes, as do *clr4Δ* mutants whose outer centromeres cannot recruit the HP1-like factor Swi6. The CENP-A^Cnp1^ signals associated with such laggards are frequently stretched or even split into two domains. Crucially, quantification of the DAPI fluorescence of individual lagging chromosomes marked by GFP suggests that these stretched/split CENP-A^Cnp1^ signals arise from individual kinetochores. We suggest that the stretching is caused by microtubule forces exerted on individual kinetochores being pulled in two directions. The phenomenon is reminiscent of the morphology of merotelically attached kinetochores observed in mammalian, plant, and insect cells [2, 25, 26].

**Figure 7:**
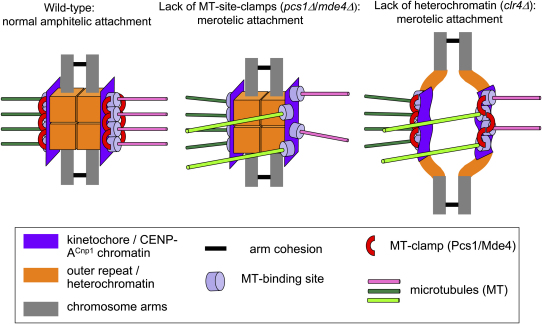
A Model Suggesting Role of Pcs1/Mde4 and Centromeric Heterochromatin in Kinetochore-Microtubule Attachment We propose that the Pcs1/Mde4 complex acts in the central kinetochore domain to clamp microtubule binding sites together, that centromeric heterochromatin coating the flanking domains provide rigidity, and that both systems contribute to the prevention of merotelic attachment.

Our finding that Clr4 as well as Pcs1/Mde4 is required to suppress lagging chromatids suggests that merotelic attachment is prevented by at least two mechanisms in fission yeast: (1) Pcs1/Mde4-dependent clamps that lock together neighboring microtubule attachment sites at each single kinetochore and (2) a Clr4/Swi6-dependent centromeric heterochromatin structure that promotes amphitelic orientation. It is known that heterochromatin mutants display premature separation of sister centromeres because of a lack of cohesin at centromeres [28, 29]; the lack of geometric constraint between sister kinetochores may increase the likelihood of merotelic as well as syntelic orientations because of rotational flexibility of one kinetochore relative to its sister [6]. In addition, we suggest that heterochromatin contributes to a higher order structure (independent of the putative Pcs1/Mde4 microtubule clamps) that provides rigidity to each individual centromere/kinetochore and that this ensures that microtubule binding sites can be properly oriented. Absence of centromeric heterochromatin might cause increased kinetochore flexibility, making it more prone to interaction with microtubules coming from opposite poles. Our proposal that microtubule clamps and heterochromatin act to counter merotely in different ways is supported by our previous finding that *pcs1Δ* and *swi6Δ* mutations are synthetically lethal, which suggests that the Pcs1/Mde4 complex and centromeric heterochromatin proteins have separate functions but contribute to the same process [10]. It is also consistent with the distinct locations of putative microtubule clamps, which reside at the central kinetochore domain, and of heterochromatin, which coats the centromeric outer repeat regions. We envisage a structure in which pericentric chromatin underlies the kinetochore, forming a rigid structure against which the Pcs1/Mde4 microtubule binding site clamps can brace (Figure 7).

In mammalian cells, merotelically oriented kinetochores are not detected by the mitotic spindle checkpoint [7]. Furthermore, the spindle checkpoint protein Bub1 is not recruited to the kinetochores of lagging chromosomes and there is no evidence for the involvement of the spindle checkpoint in slowing the rate of spindle elongation in *S. pombe* mutants with lagging chromosomes [11, 28]. However, it remains to be tested whether the onset of anaphase is delayed and whether the spindle checkpoint is activated in *pcs1, mde4*, and *clr4* mutants.

As in fission yeast, kinetochores in higher eukaryotes bind multiple microtubules and therefore must ensure that all microtubule attachment sites on a single kinetochore face the same pole. Given that the function of the Csm1/Lrs4 complex is conserved in two distant yeast species, it is likely that a similar complex is employed to prevent merotelic attachment in higher eukaryotes. Moreover, it is possible that centromeric heterochromatin, which underlies vertebrate kinetochores, plays a similar role in orienting and organizing multiple microtubule binding sites.

## Supplemental Data

Document S1. Ten Figures, Four Tables, and Experimental Procedures

Movie S1. Live Analysis of Lagging Kinetochores in *clr4Δ* Cells *clr4Δ* cells expressing Nuf2-GFP were filmed. Selected frames are shown in Figure 6B.

Movie S2. Live Analysis of Lagging Kinetochores in *pcs1Δ* Cells *pcs1Δ* cells expressing Nuf2-GFP were filmed. Selected frames are shown in Figure 6C.

Movie S3. Live Analysis of Lagging Kinetochores in *clr4Δ* Cells *clr4Δ* cells expressing Nuf2-GFP were filmed. Selected frames are shown in Figure S8A.

Movie S4. Live Analysis of Lagging Kinetochores in *clr4Δ* Cells *clr4Δ* cells expressing Nuf2-GFP were filmed. Selected frames are shown in Figure S8B.

Movie S5. Live Analysis of Lagging Kinetochores in *clr4Δ* Cells *clr4Δ* cells expressing Nuf2-GFP were filmed. Selected frames are shown in Figure S8C.

Movie S6. Live Analysis of Lagging Kinetochores in *pcs1Δ* Cells *pcs1Δ* cells expressing Nuf2-GFP were filmed. Selected frames are shown in Figure S8D.

Movie S7. Live Analysis of Lagging Kinetochores in Wild-Type Cells Wild-type cells expressing Nuf2-GFP were filmed. Selected frames are shown in Figure 6A.
