# Supplementary material for: The Kinetochore Proteins Pcs1 and Mde4 and Heterochromatin Are Required to Prevent Merotelic Orientation
Source: Curr Biol. 2007 Jul 17;17(14):1190–200. doi: 10.1016/j.cub.2007.06.044 (PMC1931489; doi:10.1016/j.cub.2007.06.044)
Supplement: Document S1. Ten Figures, Four Tables, and Experimental Procedures [file mmc1.pdf]

Supplemental Data

The Kinetochore Proteins Pcs1 and Mde4 and Heterochromatin Are Required to Prevent Merotelic Orientation

Juraj Gregan, Christian G. Riedel, Alison L. Pidoux, Yuki Katou, Cornelia Rumpf, Alexander Schleiffer, Stephen E. Kearsey, Katsuhiko Shirahige, Robin C. Allshire, and Kim Nasmyth

Supplemental Experimental Procedures

Bioinformatics

Lrs4 Database Search

By using iterative PSI-BLAST searches with the Lrs4 from *Saccharomyces cerevisiae* (Sc, ref|NP_010727.1|; masked for coiled-coil and low-complexity regions) in the nonredundant database at the National Center for Biotechnology Information (NCBI, Bethesda) [S1], we could detect one ortholog in *Candida glabrata* (Cg, ref|XP_448171.1|; round 2, e-value 0.002) and in subsequent rounds, orthologs in *Ashbya gossypii* (Ag, gb|AAS53009.1|) and *Kluyveromyces lactis* (ref|XP_451585.1|).

Mde4 Protein Sequences

The *Schizosaccharomyces pombe* Mde4 protein sequence was derived from the NCBI protein database (gi|2894279|emb|CAA17047.1), the Mde4 fragment from *Schizosaccharomyces japonicus* was translated via *S. pombe* Mde4 and genomic sequences deposited by the Whitehead Institute for Biomedical Research at the NCBI trace archive (gnl|ti|955794238 and gnl|ti|1044634558).

The motif within the coiled-coil region was defined by the Gibbs recursive motif sampler [S2].

The alignment was generated with ProbCons [S3] graphically processed with ClustalX [S4].

Tandem Affinity Purification

TAP purifications from mitotic and meiotic cells and mass spectrometry were performed as previously described [S5].

ChIP-chip Method

ChIP-chip analyses were carried out as previously described [S6] except for DNA amplification step. We noticed that PCR-based amplification of *pombe* genomic DNA is susceptible for biased amplification, because of more complex genomic sequence of *S. pombe* than *S. cerevisiae*. Therfore, we adopted T7-based in vitro transcription linear amplification method (IVT amplification method) as previously described [S7]. After addition of poly T tails to the ends of DNA fragments to be amplified by terminal transferase, strand synthesis was carried out with a T7 polyA primer adaptor to produce double-stranded templates suitable for IVT. After IVT was performed, amplification products was transformed into cDNA and used for labeling and hybridization to *S. pombe* whole-genome tiling array as previously described [S6]. *S. pombe* whole-genome tiling array was commercially available from Affymetrix (*S. pombe* 1.0FR array, P/N 900647). The *S. pombe* tiling array consists of 6 million probe pairs tiled through the complete *S. pombe* genome. Probes are tiled for both strands of the genome at an average of 20 base pair resolution, as measured from the central position of adjacent 25-mer oligos, creating an overlap of approximately 5 base pairs on adjacent probes. The information of oligo probes on the arrays (sequence and location on the chip) is available from Affymetrix. For the primary analysis of tiling chip data, a domestic software was constructed that exactly follows the statistical algorithm used for Affymetrix GeneChip Operating Software. The detailed information for the algorithm used can be downloaded from the Affymetrix web site at http://www.affymetrix.com/support/technical/technotes/statistical_reference_guide.pdf. The analysis is available from K.S. on request. In all cases, one unit for analysis (locus) was set to 100 bp with the step size of 50 bp. Fold change value, change p value, and detection p value for each locus were obtained by primary analysis. For the discrimination of positive and negative signals for the binding, we used three criteria as follows. First, the reliability of the signal strength was judged by detection p value of each locus (p value $\leq$ 0.001). Second, reliability of binding ratio was judged by change p value (p value $\leq$ 0.001). Third, clusters consisting of at least 500 bp contiguous loci that satisfied the above two criteria were selected, because it is known that a single site of protein-DNA interaction resulted in immunoprecipitation of DNA fragments that hybridized not only to the locus of the actual binding site but also to its neighbors [S6, S8].

All the array data are available at GEO database (http://www.ncbi.nlm.nih.gov/projects/geo/) through the accession number of GSE5257.

Strains, Media, and Growth Conditions

The genotypes of *S. pombe* strains used in this study are listed in the Table S4. *S. pombe* media and growth conditions were as described in [S9].

Microscopy

The immunofluorescence and microscopy techniques were as described in [S9]. Chromatin binding assay was performed as described in [S10].

Immunofluorescence and Live-Cell Imaging

Cultures were grown at 25°C and then shifted to 18°C for 2–4 hr (to increase the frequency of lagging chromosomes). Cells were fixed for 7 min with 3.7% formaldehyde at room temperature before processing for immunofluorescence [S11]. Sheep anti-Cnp1 serum was used at 1:3000; rabbit anti-GFP (Molecular Probes) was used at 1:3000. Alexa-488, -568, or -594 coupled secondary antibodies (Molecular Probes) were used at 1:1000. Live imaging was performed as described previously [S12]. Images were captured with a Zeiss Axioplan imaging 2 microscope and 100× Plan Neofluar 1.3NA, 100× PlanApochromat 1.3NA, or 100× Fluar 1.3NA objectives, Chroma 86000 and 84000 filter sets, Photometrics CoolSNAP-HQ camera, with Metamorph Software (Universal Imaging). Images presented have been autoscaled (Metamorph). Further adjustments to brightness and contrast have been made to the cen2-GFP image in Figure 5 (green channel only) to highlight the cen2-GFP spots. Filming was performed at 21°C–25°C. 1 s exposures were taken every 30 s. Quicktime movies are speeded up 180×. Brightness and contrast have been altered in the selected stills shown in Figures 6 and S8.

Classification and Measurements of Lagging Chromosomes

To determine the percentage of lagging kinetochores that were stretched versus normal, cells containing lagging chromosomes were identified in the DAPI channel, and then kinetochore and DAPI images were captured. Lagging kinetochores were classified on the basis of morphology (see Figure 4) as normal (dot-like), elongated, bilobed shape, or split. It was not possible to categorize some lagging kinetochores, e.g., because the pattern of DAPI and CENP-A^{Cnp1} staining was too complex, and these kinetochores were not included in the totals. Kinetochore dimensions were measured by Metamorph software. Kinetochores were classified as elongated if their ratio of length (in the plane of the spindle) to width was 1.5 or greater; those with a length:width ratio of less than 1.5 were classified as dot-like. Thus, the elongated class is probably underestimated; this may be reflected in the measurement data presented in Table S2, which indicates that the “dot-like” lagging kinetochores are slightly elongated in the plane of the spindle.

Quantification of DAPI Fluorescence Intensity

With the known sizes of fission yeast chromosomes [S13], the theoretical relative masses of all chromosomes (or combinations) were

A **Mitotic Pcs1-TAP MS/MS**

	Protein	description	Molecular Weight (kDa)	Mascot Score
1	Mde4	protein of unknown function	48	873
2	Ssa2	heat shock protein	70	497
3	Rps101	ribosomal protein	29	403
4	Rpl2701	ribosomal protein	15	295
5	Rps102	ribosomal protein	29	281
6	Pcs1	kinetochore protein	26	271
7	Rps13	ribosomal protein	17	229
8	Rpl35	ribosomal protein	14	212
9	Rpl8	ribosomal protein	28	210
10	Rpl402	ribosomal protein	39	198
11	Rpl15	ribosomal protein	24	190
12	Rps1702	ribosomal protein	16	186
13	Rps1802	ribosomal protein	17	181
14	Rpl1502	ribosomal protein	24	178
15	Rpl13	ribosomal protein	24	177
16	Rps1701	ribosomal protein	16	174
17	Rpl6	ribosomal protein	21	174
18	Rpl1901	ribosomal protein	23	174
19	Rpl26	ribosomal protein	14	170
20	Rps1101	ribosomal protein	16	165
21	Rpl3601	ribosomal protein	11	155
22	Tdh1	glyceraldehyde-3-phosphate dehydrogenase	36	153
23	Rps802	ribosomal protein	23	152
24	Rpl14	ribosomal protein	15	150
25	Rpl1801	ribosomal protein	21	133

B **Meiotic Pcs1-TAP MS/MS**

	Protein	description	Molecular Weight (kDa)	Mascot Score
1	mde4	protein of unknown function	48	1469
2	ssa2	heat shock protein	70	1187
3	ssa1	heat shock protein	70	915
4	clp1	Cdc14-related protein phosphatase	61	762
5	pcs1	kinetochore protein	30	688
6	tdh1	glyceraldehyde-3-phosphate dehydrogenase	36	592
7	ef1a-a	translation elongation factor	50	546
8	crp79	poly(A) binding protein	82	441
9	gpd3	glyceraldehyde 3-phosphate dehydrogenase	36	437
10	rps102	ribosomal protein	29	435
11	bip1	heat shock protein	73	425
12	sks2	heat shock protein	67	415
13	rps101	ribosomal protein	29	388
14	rpl402	ribosomal protein	40	374
15	rpl401	ribosomal protein	40	361
16	rpl301	ribosomal protein	44	359
17	tif471	translation initiation factor	154	342
18	rps601	ribosomal protein	28	315
19	rpl8	ribosomal protein	28	294
20	rps13	ribosomal protein	17	282
21	rpl2302	ribosomal protein	15	275
22	rpl402	ribosomal protein	39	270
23	rpl1902	ribosomal protein	22	247
24	rpl26	ribosomal protein	14	233
25	rpl2701	ribosomal protein	15	225

Figure S1. Full List of Proteins Identified by Tandem Mass Spectrometry Copurifying with TAP-Tagged Pcs1

(A) Proteins associated with TAP-tagged Pcs1 were isolated by tandem affinity purification from cycling mitotic *S. pombe* cells (K12253).

(B) Proteins associated with TAP-tagged Pcs1 were isolated by tandem affinity purification from diploid *S. pombe* cells induced to enter synchronous meiosis by inactivation of Pat1 (K12524) [S14] and harvested around metaphase I.

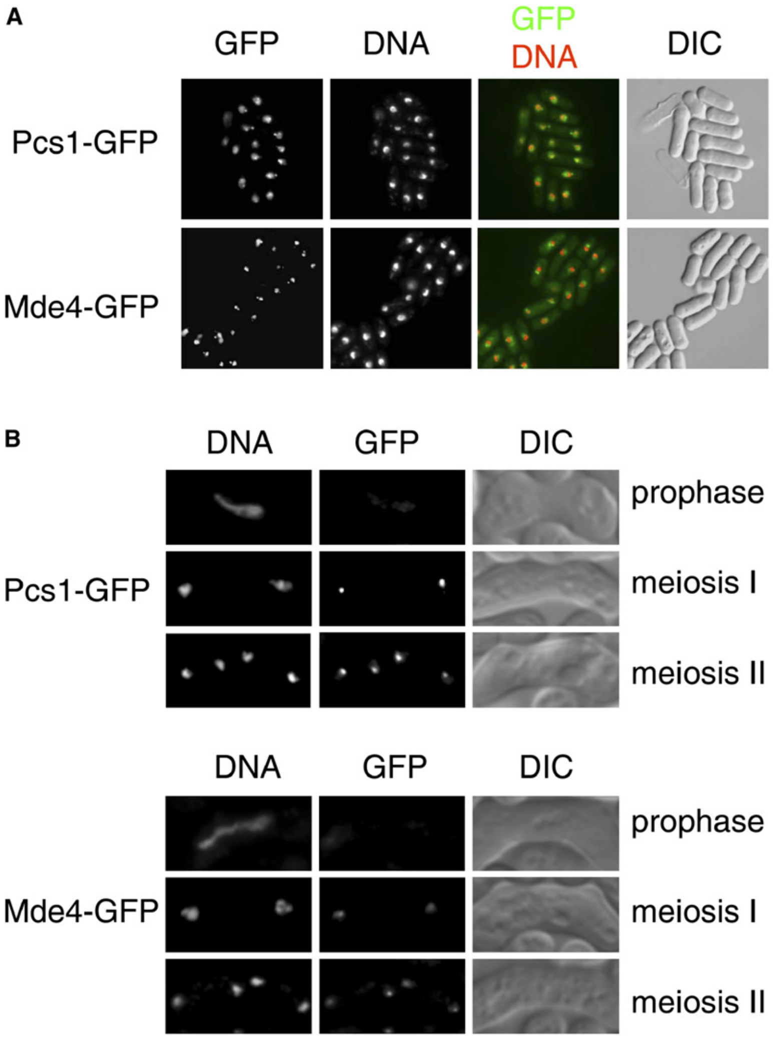

Figure S2. Characterization of Pcs1 and Mde4 Localization during Mitosis and Meiosis

(A) Cycling *S. pombe* cells expressing Pcs1-GFP (K11251) or Mde4-GFP (K12417) were fixed, stained with DAPI, and analyzed by fluorescence microscopy.

(B) *S. pombe* h^{90} cells expressing Pcs1-GFP (K11251) or Mde4-GFP (K12417) were sporulated, fixed, stained with Hoechst, and analyzed by fluorescence microscopy.

calculated and expressed as percentages of the total DNA in a mitotic cell (2n DNA content), as shown in Table S3. Images of cells with lagging chromosomes were inspected and those that fit the following criteria analyzed: the laggard was distinct from the other DNA, and the DNA at both poles was also in focus. Cells with very complex segregation patterns were not included in the analysis. Rare cells (~1%–5% of cells with laggards) with very small DAPI-stained objects between the poles that might represent chromosome fragments were not included. Quantification of DAPI fluorescence intensity was carried out with Metamorph software. Regions of interest (ROI) were drawn around each DAPI-stained object in the image (typically three), and identical ROIs were placed at DAPI-negative regions of the image to measure background fluorescence (i.e., 3 DNA ROIs and 3 background ROIs per image). For each DNA ROI, DAPI fluorescence intensity was integrated, and the appropriate integrated background fluorescence was subtracted. The relative mass of each DAPI object was expressed as a percentage of the total DAPI fluorescence in the cell. Three sets of cells were analyzed. (1) The method was shown to be valid and accurate by analysis of *clr4Δ* cen2-GFP cells with single chromosome 2 laggards (i.e., one cen2-GFP spot on the laggard, one at the pole). The theoretical relative masses are: 50% (chromosomes 1+2+3 at one pole): 33.4% (chromosomes 1+3 at the other pole): 16.7% (chromosome 2 laggard). The relative masses calculated from DAPI fluorescence intensity measurements (49.0% ± 2.1%: 33.0% ± 2.2%: 17.8% ± 1.6%, n = 16) were very similar to theoretical values. In addition, three cells in which the cen2-GFP spots were very close or coincident (i.e., adjacent or unseparated chromosome 2) were analyzed. (2) *clr4Δ* and *pcs1Δ* cells with cen2-GFP that were stained with anti-CENP-A^{Cnp1} antibodies. Because it was critical to establish that the laggards with stretched/split CENP-A^{Cnp1} morphology are

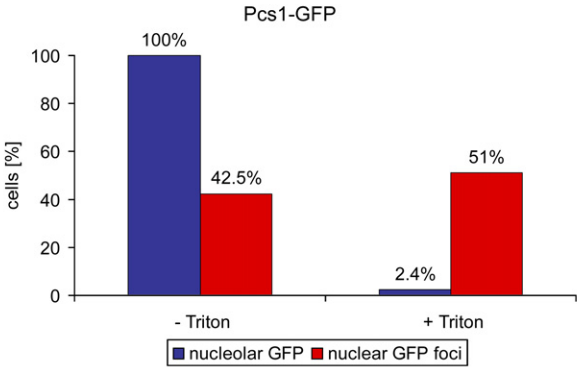

Figure S3. Association of Pcs1 with Chromatin

Asynchronous log phase cells expressing Pcs1-GFP (K11251) were permeabilized by zymolyase digestion and either directly fixed (−Triton) or extracted with detergent and then fixed (+Triton). Graphs show the percentage of cells with nucleolar Pcs1-GFP and cells with nuclear Pcs1-GFP foci.

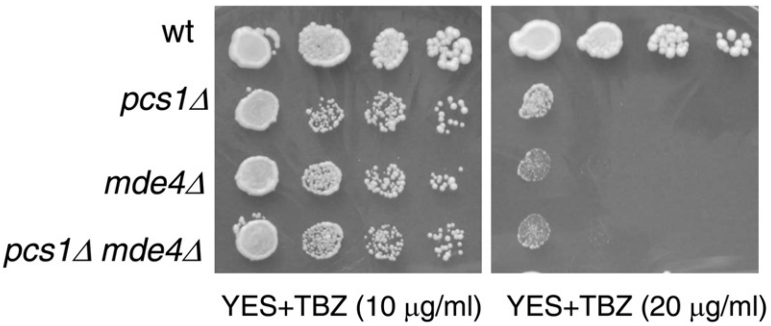

Figure S4. *pcs1Δ* and *mde4Δ* Mutants Are Sensitive to Thiabendazole

Wild-type (K11248), *pcs1Δ* (K14820), *mde4Δ* (K14818), and *pcs1Δ mde4Δ* (K14822) cells were serially diluted and spotted onto YES medium containing 10 or 20 μg/ml of thiabendazole (TBZ). Cells were grown for 2 days at 32°C.

indeed single chromatids, the analysis focused on these cells. Therefore, only a small number of cells displaying a “dot” CENP-A^{Cnp1} signal on laggards were analyzed for the purposes of comparison (although they account for ~70% of cells with lagging chromosomes). Cells with unseparated/adjacent cen2-GFP were also analyzed. (3) *clr4Δ*, *pcs1Δ*, and *mde4Δ* cells with unmarked chromosomes (cells used for Figure 4 and Tables S1 and S2). All cells with split/stretched CENP-A^{Cnp1} morphology that were suitable for DAPI fluorescence quantification (see above) were analyzed. A few cells with a large lagging mass of DNA, which had been interpreted as two adjacent laggards (e.g., “dot” plus “stretched” laggards), were also analyzed.

Supplemental References

S1. Altschul, S.F., and Koonin, E.V. (1998). Iterated profile searches with PSI-BLAST—a tool for discovery in protein databases. Trends Biochem. Sci. *23*, 444–447.

S2. Thompson, W., Rouchka, E.C., and Lawrence, C.E. (2003). Gibbs recursive sampler: finding transcription factor binding sites. Nucleic Acids Res. *31*, 3580–3585.

S3. Do, C.B., Mahabhashyam, M.S., Brudno, M., and Batzoglou, S. (2005). ProbCons: probabilistic consistency-based multiple sequence alignment. Genome Res. *15*, 330–340.

S4. Chenna, R., Sugawara, H., Koike, T., Lopez, R., Gibson, T.J., Higgins, D.G., and Thompson, J.D. (2003). Multiple sequence alignment with the Clustal series of programs. Nucleic Acids Res. *31*, 3497–3500.

S5. Riedel, C.G., Katis, V.L., Katou, Y., Mori, S., Itoh, T., Helmhart, W., Galova, M., Petronczki, M., Gregan, J., Cetin, B., et al. (2006). Protein phosphatase 2A protects centromeric sister chromatid cohesion during meiosis I. Nature *441*, 53–61.

S6. Katou, Y., Kanoh, Y., Bando, M., Noguchi, H., Tanaka, H., Ashikari, T., Sugimoto, K., and Shirahige, K. (2003). S-phase checkpoint proteins Tof1 and Mrc1 form a stable replication-pausing complex. Nature *424*, 1078–1083.

S7. Katou, Y., Kaneshiro, K., Aburatani, H., and Shirahige, K. (2006). Genomic approach for the understanding of dynamic aspect of chromosome behavior. Methods Enzymol. *409*, 389–410.

S8. Lindroos, H.B., Strom, L., Itoh, T., Katou, Y., Shirahige, K., and Sjogren, C. (2006). Chromosomal association of the Smc5/6 complex reveals that it functions in differently regulated pathways. Mol. Cell *22*, 755–767.

S9. Rabitsch, K.P., Petronczki, M., Javerzat, J.P., Genier, S., Chwalla, B., Schleiffer, A., Tanaka, T.U., and Nasmyth, K. (2003). Kinetochore recruitment of two nucleolar proteins is required for homolog segregation in meiosis I. Dev. Cell *4*, 535–548.

S10. Gregan, J., Lindner, K., Brimage, L., Franklin, R., Namdar, M., Hart, E.A., Aves, S.J., and Kearsey, S.E. (2003). Fission yeast Cdc23/Mcm10 functions after pre-replicative complex formation to promote Cdc45 chromatin binding. Mol. Biol. Cell *14*, 3876–3887.

S11. Pidoux, A.L., Richardson, W., and Allshire, R.C. (2003). Sim4: a novel fission yeast kinetochore protein required for centromeric silencing and chromosome segregation. J. Cell Biol. *161*, 295–307.

S12. Pidoux, A.L., Uzawa, S., Perry, P.E., Cande, W.Z., and Allshire, R.C. (2000). Live analysis of lagging chromosomes during anaphase and their effect on spindle elongation rate in fission yeast. J. Cell Sci. *113*, 4177–4191.

S13. Wood, V., Gwilliam, R., Rajandream, M.A., Lyne, M., Lyne, R., Stewart, A., Sgouros, J., Peat, N., Hayles, J., Baker, S., et al. (2002). The genome sequence of *Schizosaccharomyces pombe*. Nature *415*, 871–880.

S14. Yamamoto, M. (1996). The molecular control mechanisms of meiosis in fission yeast. Trends Biochem. Sci. *21*, 18–22.

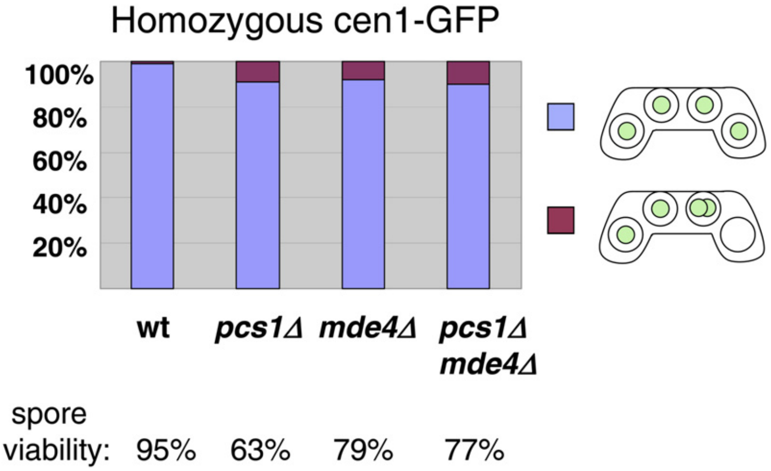

Figure S5. Pcs1 and Mde4 Are Dispensable for Chromosome Segregation during Meiosis I but Are Required for Meiosis II

A wild-type h^{90} *lys1-GFP* strain carrying *lacO* sequences integrated near the *lys1* locus and expressing the LacI-GFP fusion protein, which binds to *lacO* (wt) (K11248), and h^{90} *lys1-GFP* strains carrying either the knockout allele of *pcs1* (*pcs1Δ*) (K11420), or the knockout allele of *mde4* (*mde4Δ*) (K12495), or a double knockout (*pcs1Δ mde4Δ*) (K12539) were sporulated. Unfixed cells were stained with Hoechst and examined under the fluorescence microscope. Segregation of chromosome I was scored in at least 80 asci. Spore viability was determined by dissecting spores from at least 60 asci and scoring the ability to form colonies.

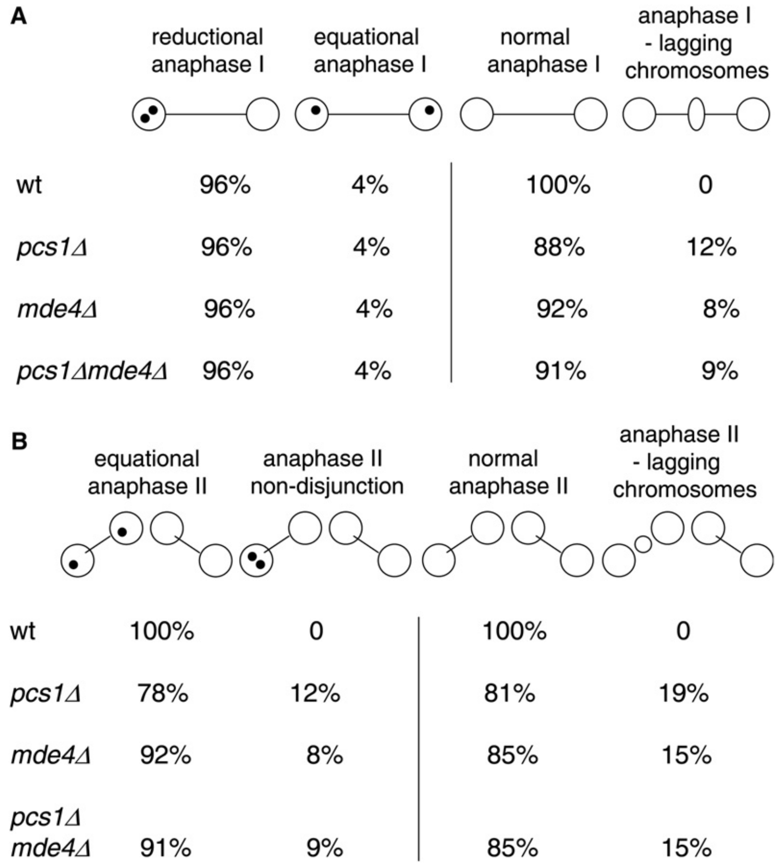

Figure S6. Analysis of Sister-Chromatid Segregation during Meiosis I and Meiosis II

(A) The wild-type strain h^- *lys1*-GFP (wt) (K11338) or strain h^- *lys1*-GFP strain carrying a knockout allele of *pcs1* (K14820), *mde4* (K14818), or a double knockout of *pcs1* and *mde4* (K14822) were crossed to h^+ strains that were of the same genotype and lacked *lys1*-GFP (K11339, K14821, K14819, and K14823, respectively). Cells were sporulated, fixed, stained with Hoechst and antibodies against tubulin and GFP, and examined under the fluorescence microscope. Chromosome I was visualized by *lys1*-GFP (heterozygous *lys1*-GFP). Anaphase I cells were identified according to tubulin staining of the spindle and number of nuclei. At least 100 cells were counted. Segregation of chromosome I and lagging chromosomes were scored in at least 100 anaphase I cells.

(B) Cells were processed and analyzed as described in (A). Segregation of chromosome I, visualized by *lys1*-GFP (heterozygous *lys1*-GFP), and lagging chromosomes were scored in at least 90 anaphase II cells.

Table S1. Morphology of CENP-A^{Cnp1} Staining on Lagging Chromosomes

Strain	Dot	Split	Bilobed	Elongated	Total Stretched
clr4Δ (n = 60)	75	10	11.7	3.3	25
pcs1Δ (n = 117)	73	12.8	6	8.5	27
mde4Δ (n = 91)	68	16.5	11	4.4	32

The CENP-A^{Cnp1} staining pattern on lagging chromosomes was classified as dot-like (i.e., of normal appearance), split, bilobed, or elongated, examples of which are shown in Figure 4. Numbers are given as percentages. Ambiguous cases that could not be classified were excluded from the analysis. The elongated class is composed of kinetochores for which the ratio of length (along the spindle axis) to width was at least 1.5; those with a length:width ratio of less than 1.5 were included in the "dot" category.

Table S2. Dimensions of CENP-A^{Cnp1}-Stained Kinetochores

Strain	Dot-Shaped Kinetochores	Stretched Kinetochores
Wild-type	0.42 ± 0.03	not applicable
	0.40 ± 0.03 (n = 34)	
clr4Δ	0.48 ± 0.06	0.75 ± 0.15
	0.43 ± 0.04 (n = 12)	0.40 ± 0.03 (n = 14)
pcs1Δ	0.47 ± 0.06	0.78 ± 0.19
	0.40 ± 0.05 (n = 15)	0.40 ± 0.03 (n = 29)
mde4Δ	046 ± 0.05	0.85 ± 0.16
	0.41 ± 0.04 (n = 34)	0.40 ± 0.04 (n = 29)

CENP-A^{Cnp1}-stained kinetochores were measured in the indicated strains. Measurements are in microns (mean ± SD): top numbers indicate length of kinetochore (i.e., along plane of spindle); lower numbers indicate width. Lagging kinetochores in anaphase were measured, except for wild-type in which kinetochores in early mitosis were measured.

Table S3. Contribution of Individual Chromosomes and Their Combinations to the Total Chromosomal DNA

Chromosome	Size	Contribution to 2n DNA
chr1+chr2+chr3	13.8 Mb (1n)	50%
chr1	5.7 Mb	20.7%
chr2	4.6 Mb	16.7%
chr3	3.5 Mb	12.7%
chr1+chr1		41.4%
chr2+chr2		33.4%
chr3+chr3		25.4%
chr1+chr2		37.4%
chr1+chr3		33.4%
chr2+chr3		29.4%

The sizes of *S. pombe* chromosomes are shown, along with the percentage contribution each chromosome or combination of chromosomes makes to the 2n DNA content of a mitotic cell.

Table S4. Strain List

Strains	Genotype
K12253	*h^{90} PCS1-HA-TAP::kanMX*
K12524	*h^- pat1-114 PCS1-HA-TAP::kanMX ade6-M216*
	h^- pat1-114 PCS1-HA-TAP::kanMX ade6-M210
K11251	*h^{90} pcs1-GFP::LEU2 leu1 ura4 ade6-210 his1-102*
K12417	*h^{90} MDE4-GFP::kanMX*
K13893	*h^{90} swi6-GFP::kan leu1 ade6-M216*
K12111	*h^{90} sgo2-GFP-kanMX ura4*
K11338	*h^- LacO-lys1+ GFP-LacI-his7+ leu1 ura4 ade6-210*
K14820	*h^- LacO-lys1+ GFP-LacI-his7+ leu1 ura4 ade6-210 pcs1::ClonNat*
K14818	*h^- LacO-lys1+ GFP-LacI-his7+ leu1 ura4 ade6-210 mde4::kanMX*
K14822	*h^- LacO-lys1+ GFP-LacI-his7+ leu1 ura4 ade6-210 mde4::kanMX pcs1::ClonNat*
K11339	*h^+ lys1 his7 leu1 ura4 ade6-210*
K14821	*h^+ lys1 his7 leu1 ura4 ade6-210 pcs1::ClonNat*
K14819	*h^+ lys1 his7 leu1 ura4 ade6-210 mde4::kanMX*
K14823	*h^+ lys1 his7 leu1 ura4 ade6-210 pcs1::ClonNat mde4::kanMX*
K11248	*h^{90} leu1 ura4 LacO-lys1+ GFP-LacI-his7+*
K11420	*pcs1::ClonNat h90 cen1-GFP leu1- ura4-*
K12495	*h^{90} leu1 ura4 LacO-lys1+ GFP-LacI-his7+ mde4::kanMX*
K12539	*h^{90} del-pcs1::URA4 leu1 ura4 LacO-lys1+ GFP-LacI-his7+ mde4::kanMX*
K11248	*h^{90} leu1 ura4 LacO-lys1+ GFP-LacI-his7+*
RA1645	*h^+ ade6-210 leu1-32 ura4-D18 arg3-D4 his3-D1*
RA2268	*h^+ clr4Δ::$ura4^+$ ade6-210 leu1-32 ura4-D18 otr1R(SphI):ade6*
RA6289	*h clr4Δ::$LEU2^+$ nuf2-GFP-KAN leu1-32*
RA6370	*h^+ pcs1Δ::$ura4^+$ leu1 ura4 ade6-210 lys1 his7*
RA6419	*pcs1Δ::$ura4^+$ mis12-GFP-$LEU2^+$ leu1-32 ade6-210 ura4*
RA6428	*pcs1Δ::$ura4^+$ nuf2-GFP-KAN leu1-32 ade6-210 ura4*
RA8618 = K14819	*h^+ mde4Δ::KANMX4 lys1 his7 leu1 ura4 ade6-210*
FY10533	*pcs1Δ::ClonNat cen2(D107)::KAN-$ura4^+$-lacO $his7^+$::lacI-GFP*

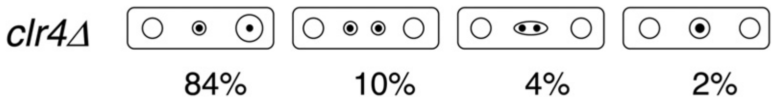

Figure S7. Lagging Chromosomes in *clr4Δ* Cells Are Single Chromatids

clr4Δ cells in which *cen2* is marked by GFP were fixed and processed for immunofluorescence with anti-GFP antibody. Those with a lagging *cen2*-GFP signal were scored (n = 100).

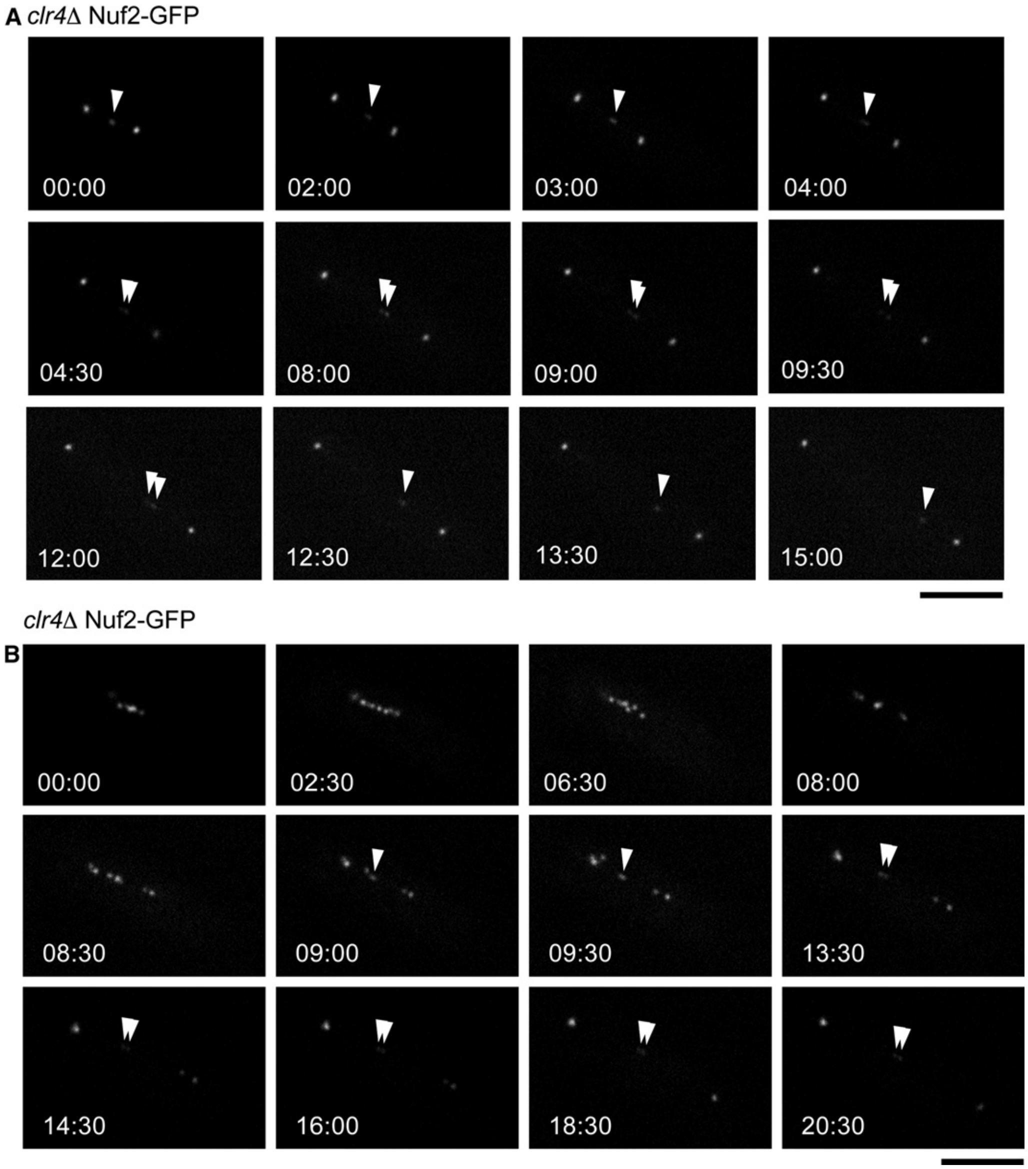

Figure S8 (*continued*).

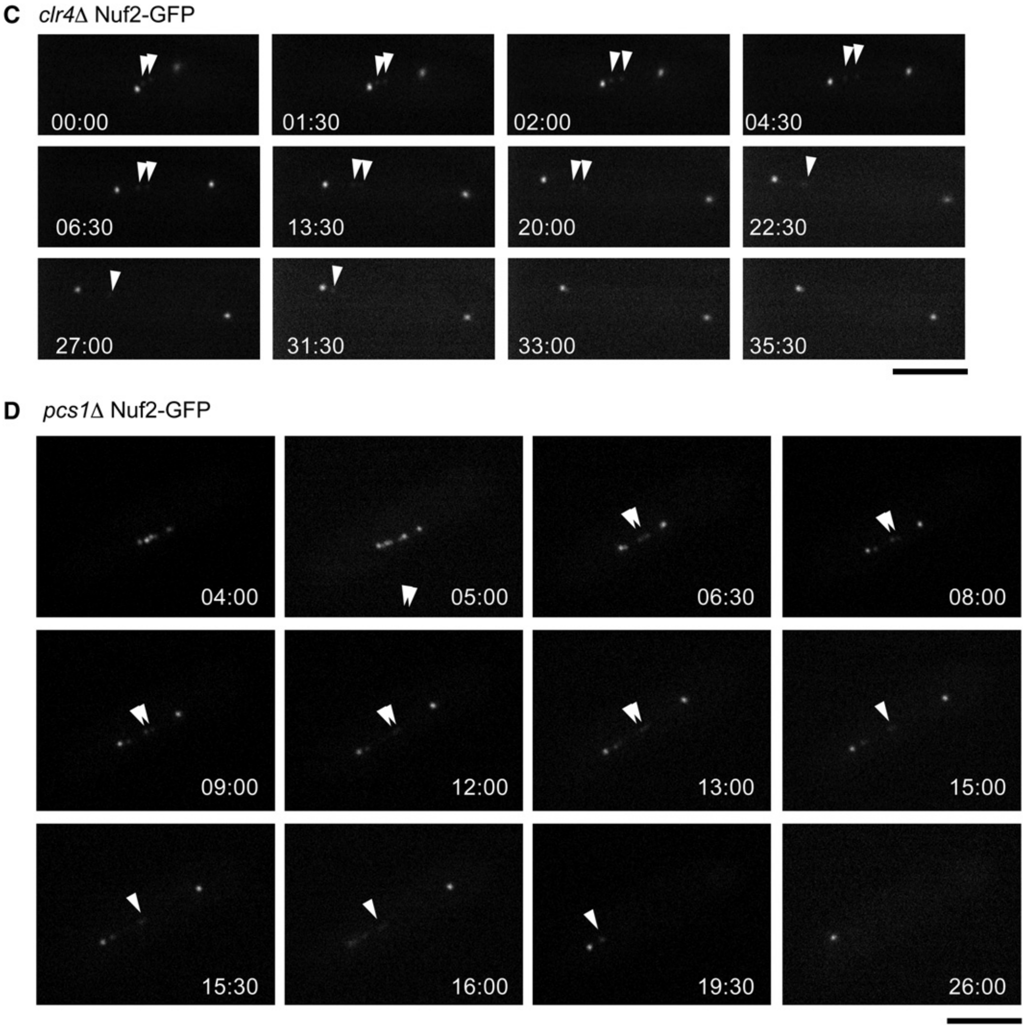

Figure S8. Live Analysis of Lagging Kinetochores

clr4Δ (A, B, C) or *pcs1Δ* (D) cells expressing Nuf2-GFP were filmed (Movies S3–S6). Selected frames are shown; times indicated are in minutes. Arrowheads indicate positions of lagging kinetochores: single arrowhead indicates a dot-shaped or elongated kinetochore; double arrowhead indicates a split kinetochore. Scale bars represent 5 μm.

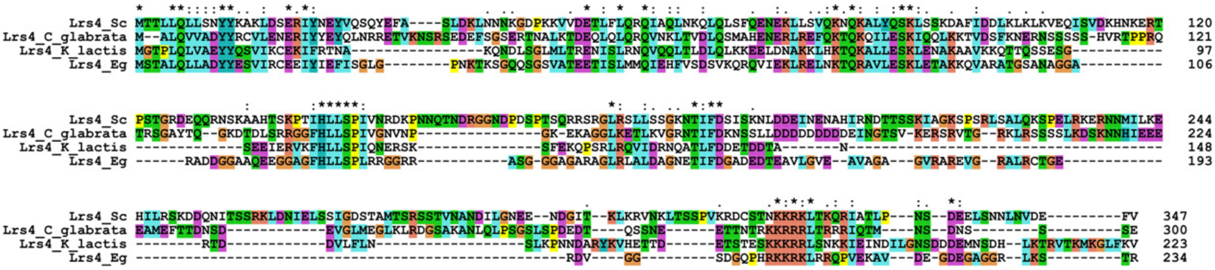

Figure S9. Multiple Sequence Alignment of Lrs4 Proteins

The alignment was generated with ProbCons [S3] graphically processed with ClustalX [S4].

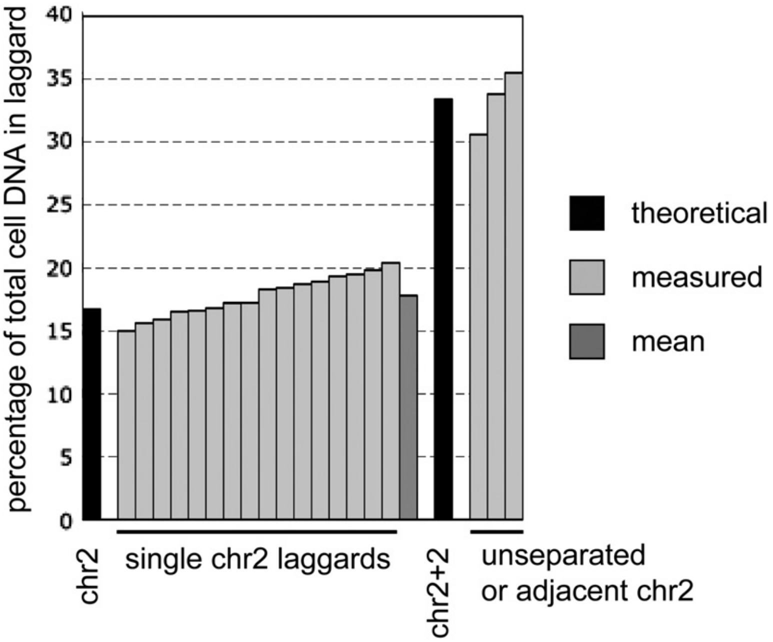

Figure S10. DAPI Fluorescence Quantification of Chromosome II Laggards Marked with cen2-GFP in *clr4Δ* Mutant
